# The Plant Interspecific Association in the Revegetated Alpine Grasslands Determines the Productivity Stability of Plant Community Across Restoration Time on Qinghai–Tibetan Plateau

**DOI:** 10.3389/fpls.2022.850854

**Published:** 2022-03-21

**Authors:** Shengnan Wu, Lu Wen, Shikui Dong, Xiaoxia Gao, Yudan Xu, Shuai Li, Quanming Dong, Kelly Wessell

**Affiliations:** ^1^School of Grassland Science, Beijing Forestry University, Beijing, China; ^2^Ministry of Education Key Laboratory of Ecology and Resource Use of the Mongolian Plateau and Inner Mongolia Key Laboratory of Grassland Ecology, School of Ecology and Environment, Inner Mongolia University, Hohhot, China; ^3^State Key Laboratory of Vegetation and Environmental Change, Institute of Botany, Chinese Academy of Sciences, Beijing, China; ^4^College of Grassland Science, Shanxi Agricultural University, Jinzhong, China; ^5^College of Resources and Environment, Shanxi Agricultural University, Jinzhong, China; ^6^Qinghai Academy of Animal and Veterinary Sciences, Qinghai University, Xining, China; ^7^Tompkins Cortland Community College, Dryden, NY, United States

**Keywords:** cultivated grasslands, stability, interspecific association, Qinghai–Tibet Plateau, restoration

## Abstract

Grassland cultivation is the key measure for restoring “Black Beach,” the extremely degraded alpine meadow in the Three River Headwater Area of the Qinghai–Tibetan Plateau. In this study, we examined the inter-specific relationship in the vegetation community of cultivated grasslands with different restoration times through the network analysis method. The results showed that with the extension of restoration time, the development of cultivated grassland would lead to increasing neutral interactions among the plant species. The proportion of species with positive and negative associations in the community decreased, while the number of species-independent pairs increased significantly. The complexity of plant interspecific association (species network density) had more influence on community stability with the extension of recovery time, which can be used to quantify the characteristics of community structure.

## Introduction

At present, grassland ecosystems, the typical human-managed ecosystem through grazing, are the focus of terrestrial ecosystems studies in the field of ecosystem function of biodiversity (Isbell, 2010). On the Qinghai–Tibetan Plateau (QTP), the “Third Pole of Earth,” alpine grassland is the largest ecosystem and provides critically important functions and services such as water regulation, carbon sequestration, biodiversity conservation, livestock production, and pastoral culture (Dong et al., 2013; Liu B. et al., 2018; Xu et al., 2019). However, natural and human factors such as climate change and livestock overgrazing have resulted in massive grassland degradation on the QTP (Zeng et al., 2015). In the past decade, approximately 26% of the alpine grasslands in the Three River Headwater Area in the central QTP have been severely degraded as “Black Beach” or “Black Soil Land,” which is defined as the bare land in winter and the sparsely covered land with annual weeds or poisonous plants in summer (Li et al., 2014). Caused mainly by overgrazing, “Black Beach” is an area that has little grazing value and degraded ecosystem services (Dong et al., 2013; Shang et al., 2018). Grassland degradation has not only dramatically reduced ecosystem service functions, but also seriously affected the social adaptability of local herdsmen (Liu S.B. et al., 2018; Shen et al., 2019), limiting sustainable development of these coupled human–natural systems at local and regional levels (Dong et al., 2012). To alleviate the negative effects of grassland degradation on natural ecosystems and social–economic systems, governments and researchers of various countries have made large-scale efforts to restore degraded grassland. Developing cultivated grasslands is one of the effective measures to alleviate the serious degradation of alpine grasslands (Wang et al., 2015). A large number of experimental studies have shown that the establishment of artificial vegetation is one of the quick and effective ways to restore the production and ecological functions of extremely degraded alpine meadow, but this method also has the problem of re-degradation, so it is necessary to fully understand the restoration of artificial grassland habitat and explore suitable artificial grassland reconstruction techniques (Feng et al., 2010; Shang et al., 2018).

The relationship between stability and biodiversity has been the focus of much ecological research and is key to understanding trends in global biodiversity (Isbell et al., 2009; Liu B. et al., 2018); however, the explanatory model and mechanism of the relationship between stability and biodiversity are still controversial (Loreau and De Mazancourt, 2013). Species play a vital role in the ecosystem, as well as in the function of the ecosystem (Cleland, 2011). Previous studies exploring the relationship between diversity and stability have emphasized the important role of species, functional group composition, and inter-specific relationships in productivity (Klanderud, 2010; Wang et al., 2013). Isbell et al. (2009) proposed that the interaction between species could promote biodiversity and stability. Recently, worldwide scientists have begun to use networks to help analyze ecological consequences of various types of species interactions (Zhou et al., 2011). According to Pellissier et al. (2018), it is necessary to study species composition and their interactions in the form of a network. Little is known about plant interspecific association and its relationship with community stability. However, a few scholars have recorded the stability of plant productivity and its relationships with species composition and interactions during the long-term recovery of grasslands on the QTP. Plant productivity is crucial to the restoration and sustainable management of grassland ecosystems (Maselli et al., 2013; Pallett et al., 2016; Yang et al., 2018; Zeeman et al., 2019). Species composition and functional group representation are the key factors to affect the aboveground or belowground biomass of the community (Cortois et al., 2017). For a long time, scholars have paid more attention to the effects of nutrients such as nitrogen, phosphorus, or organic carbon concentration on the plant community structure and productivity (Byrne and Jones, 2002; Zhao et al., 2018). Although some scholars have documented the changes in the diversity and stability of cultivated grassland with different restoration years (Li et al., 2014), a few researchers have examined the species composition and interactions on the stability of the artificial grassland plant community. In the field of succession research, it is often emphasized that the dynamics of plant community composition and structures related to species interactions should be studied (Bai et al., 2004). However, a few scholars have studied how the stability of alpine grassland productivity changes with time and whether this change depends on biodiversity or community composition. Heterogeneity plays an important role in the function and processes of ecosystems at different scales, and its coefficients of variation and time repetition are indicators of the stability of ecosystems (Eisenhauer et al., 2011; Lv et al., 2020). Exploring the community structure and composition is of great significance for research on ecosystems (Johnson et al., 2008).

We hope to explore the structure and relationship of the ecosystem through as many methods and indicators as possible. Social network analysis methods based on inter-specific relationships may provide a quantitative means for exploring the characteristics of community structure. Therefore, we conducted this study to examine species composition and interaction dynamics of alpine grassland communities planted on “Black Beach” during the restoration process and its influence on grassland ecosystem stability.

The objectives of this study are to address the following questions: (1) How do plant species composition and diversity of cultivated grassland change in the restoration time? (2) How do plant inter-specific relationships change in the cultivated grasslands following restoration? (3) How do the plant species diversity and the complexity of plant interspecific association affect the plant stability of cultivated grasslands in the restoration process?

## Materials and Methods

### Study Site

**FIGURE 1:**
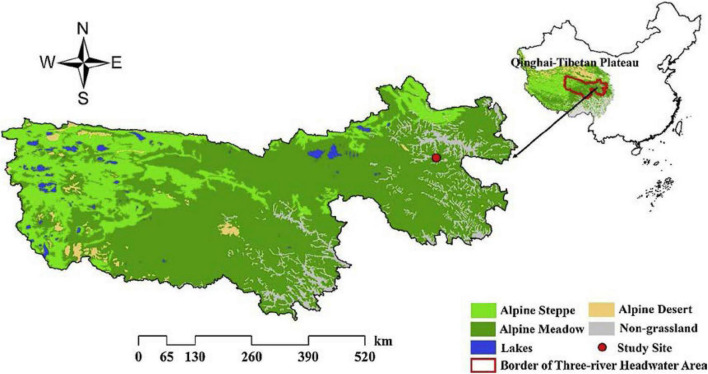
Distribution of the study sites in the three river headwater area of Qinghai–Tibetan Plateau (Gao et al., 2019).

The study site is located in the town of Dawu, Maqin County of Qinghai Province, China, the core zone of the Three Rivers Headwater Area (Figure 1). The geographical position of the study site is 32^°^31′–35°37′N in latitude, 96°54′–101°51′E in longitude, and the average elevation is over 4,000 m. The annual average temperature is 0.54°C (from 1997 to 2017 statistics) and the annual average precipitation is 518.2 mm (The coefficient of variation of the inter-divisional dynamics is 24%). The average temperature in the coldest month (January) is −11.1°C, and the average temperature in the hottest month (July) is 10.7°C. The soil type is loam (Gao et al., 2019). The primary vegetation type in the study area is an alpine meadow with *Kobresia pgymaea* as the dominant species, and *Kobresia humilis*, *Stipa aliena*, *Festuca ovina*, *Poa pratensis*, *Elymus nutans*, and *Leontopodium nanum*, as companion species. Harsh environments make alpine meadow habitats sensitive and fragile to climate change and human disturbances. Around one-quarter of the alpine meadow in the study sites have been degraded as “Black Beach” with severe soil erosion due to lowered coverage of pioneer plants such as *Aconitum flavum*, *Ligularia virgaurea*, *Pedicularis kansuensis*, and *Potentilla anserina* (Shang et al., 2018).

The severely degraded alpine meadow, “Black Beach” has been largely rebuilt with revegetated grasslands. Cultivated grassland (also called artificial grassland) is a new grassland community formed by planting one or more plants on the completely degraded grassland by using comprehensive agricultural technology. Over past decades, large areas of the “Black Beach” degraded grasslands have been restored with cultivated grassland under the governmental programs “Returning Grazing Land into Grassland” and “Grazing Ban” (Dong et al., 2013). With the support of a series of these restoration projects, numerous demonstration sites of cultivated grasslands with different restoration times have been established in the study area (Gao et al., 2019).

According to relevant information and as reported in the literature, in this study, we did the samplings based on the chronosequence/space-for-time approach in the cultivated grasslands, which were established in 2000 (A1), 2002 (A3), 2004 (A4), 2006 (A6), 2008 (A8), 2010 (A10), 2012 (A12), 2014 (A14), 2015 (A16), and 2017 (A18) and severely degraded alpine meadow (Black Beach, A0), and non-degraded healthy alpine meadow (ND). All the sampling plots in this study were located in the same demonstration study sample area accompanied by similar topography and geographical location. The planting area of each cultivated grassland was 50 hm^2^. The cultivated grasslands by sowing *E. nutans* were all degraded grasslands on “Black Beach” and were built through deep turning, harrowing, fertilizing [45 kg/hm^2^ of diammonium phosphate (NH_3_HPO_4_)], sowing (sowing amount was 45 kg/hm^2^ local seed), covering soil, crushing, fencing, and other agronomic measures.

### Field Survey

In mid-August 2017, five quadrats with an area of 1 m × 1 m were randomly selected from each plot for plant sampling and species identification. According to the classification method on the functional groups of alpine grassland plants (Wang et al., 2004), and in combination with the morphological and physiological characteristics of alpine grassland plants, we divided the species into four functional groups: Grass, Cyperaceae, Leguminosae, and the remaining species were classified as Forb (Yu et al., 2017; Zhao et al., 2017). The number of species, density, height, coverage, and frequency of individual species were estimated. In each plot, one subplot in the size of 0.5 m × 0.5 m was selected for sampling aboveground plant biomass, which was brought back to the laboratory and dried in an oven at 65°C to constant weight for measurement. After plant sampling, soil core samples were taken to a depth of 20 cm in each subplot.

Based on commonly used soil properties analysis test theory in the laboratory, we conducted experiments on the measurement of the following soil properties for each soil sample: total organic carbon (TC) and nitrogen content (TN) were tested by the elemental analyzer (Euro EA 3000, Italy), available phosphorus (AP) and available potassium (AK) were tested by an Inductive Coupled Plasma Emission Spectrometer (Germany). We also quantified soil moisture.

### Data Analysis

We used the variables related to diversity, stability, and the plant interspecific association (network analysis) mentioned above for data analysis. In detail, for characterization of species diversity and dominance structure, Pielou’s evenness index (E), Shannon–Wiener diversity index (H), and Simpson’s dominance (λ) index were calculated. Associated coefficient (AC) was used to characterize the degree of association among species (Fang et al., 2012). The range of AC is from -1 to 1. The closer the AC value is to 1, the stronger the positive association between species is. The closer the AC value is to -1, the stronger the negative association between species is. When the AC value is 0, the species are completely independent. So, we classified the inter-species relationships into three trends, positive inter-species association, negative inter-species association, and random inter-species association, respectively. The calculation of the above indices was conducted in R using the “vegan” package.

Stability (PS) was calculated as the inverse coefficient of variation, which was calculated *via* the ratio of the average biomass to the standard deviation (Strecker et al., 2016; Zhou et al., 2019). The importance value (IV) of individual species was calculated *via* the formula of IV = (C′+H′+P′)/3, where IV is the important value of each species, C′ is relative coverage, H′ is relative height, and P′ is relative frequency.

The network analysis method was used to identify the species connection in the plant community. In the network, each species was considered as a node. We used the SPAA package in R to calculate the inter-species relationship matrix, which was treated as the basic data of sub-group analysis. The modular analysis was performed to build the block model and simplify through the CONCER algorithm (convergent correlation) (Xie et al., 2019). Before drawing two complete sub-networks, we obtained the simplest network diagram that maintained the integrity of the network using UCINET 6.5 software, which is a social network analysis program containing a graphical illustration tool called Net-Draw, a program used widely to obtain the simplest network diagram of the plant community (Johnson, 1987; Thomas et al., 2007). The density is an indicator of the closeness of membership and betweenness centrality represented the ability of a member to act as an intermediary, that is, to what extent a node is located in the middle of other nodes in the network (Qin and Li, 2009). In the network of vegetation communities, the density reflects the closeness of the inter-species relationship in a vegetation community. The networks of interacting species are usually described in terms of hardiness and stability (Kleyer et al., 2018). Network density (*D*) was calculated as the ratio between the total actual lines presented in the network graphics and the potential lines (Wolfe, 1995). Betweenness Centrality (C_B_) in the network was calculated *via* the formula (Wolfe, 1995):


$$C_{B}=2\,\sum_{i=1}^{g}\left[C_{B}\,\left(n^{8}\right)-C_{B}\,\left(n_{i}\right)\right]/\left[{\left(g-1\right)}^{2}-\left(g-2\right)\right],C_{B}\,\left(n_{i}\right)=\sum_{j<k}n_{i}/g_{i\,K}$$

Where *g* represents the number of nodes in the network diagram, *g*_*ik*_ represents the shortcut of member *i* to member *k*, C_B_(n_i_) is the intermediary of node n_i_, and C_B_(n*) is the maximum node intermediation. Based on this, we calculated the network density (SND) and betweenness centrality (SNB) of the plant interspecific linkage network.

### Statistical Analysis

The data were processed and analyzed using Microsoft Excel 2016, SPSS 22.0, R version 3.5.1, and R Studio version 1.1.456. Network analysis was performed using UCINET 6.5 software. Network analysis graphs were drawn with Net Draw.

We used 28 indicators including density (*D*), network indexes containing the complexity of plant interspecific association (network density: SND) and betweenness of plant interspecific linkage network (SNB), diversity indexes, which include the evenness indexes of the total community (E-T), Grass (E-G), Cyperaceae (E-C), Legume (E-L), and Forb (E-F); the diversity of whole community (H-T), Grass (H-G), Cyperaceae (H-C), Legume (H-L), and Forb (H-F); the proportional dominance of Grass (G_λ), Cyperaceae (C_λ), Legume (L_λ), and Fords(F_λ); the proportion of inter-specific associations of plant community containing positive inter-species associations (Positive); negative inter-species associations (Negative); random inter-species associations (Random); and stability indices which include the stability of the whole community (T-PS), Grass (G-PS), Cyperaceae (C-PS), Legume (L-PS), and Forb (F-PS) and soil properties containing the carbon (C) and nitrogen (N), available phosphorus (AP), available potassium (AK), and soil moisture of the soil samples.

To test the above-mentioned indicators as the influence of independent variables on the overall stability and to avoid redundancy, we used correlation analysis to filter the variables. For the same type of indicators with a correlation coefficient greater than 0.70, one of them was retained (Zhou et al., 2019). Finally, a structural equation model (SEM) was adopted to test the hypothetical model, which was established and tested using AMOS 4.0.

## Results

### Plant Community Composition and Diversity Over Restoration Time

In each plot, the species with a 100% probability of occurrence are the grass *E. nutans*, the legume *Astragalus membranaceus* var. *membranaceus*, and the Forb *Potentilla multifida*. The dominant species was *E. nutans* (an indigenous grass planted) along restoration years except for the 8th year. The sub-dominant species changed greatly with the restoration time and the range of average species number of each plot (Table 1). Compared with “Black Beach,” the species richness of cultivated grassland decreased by 9.2% in the first recovery year and increased from the second restoration year until the 8th restoration year. Over 10 years of restoration, plant species richness in the cultivated grassland tended to continuously increase. Through all restoration years, the number of species in the plant community of artificial grassland increased. Over 16 years, there was a high similarity of species composition between the cultivated grasslands at different restoration years and the non-degraded native grassland, and low similarity of species composition between the cultivated grasslands at different restoration years and the “Black Beach.”

**TABLE 1:** Important species of the plot and their important values and proportion of comprehensive dominance of species in different functional groups.

Grassland types	Important value of species			Proportion of comprehensive dominance of species in different functional groups				Species richness
				Grass (G_λ)	Cyperaceae (C_λ)	Leguminosae (L_λ)	Fords (F_λ)	
Black Soil Beach A0	*Ligularia virgaurea*	*Artemisia hedinii*	*Oplopanax elatus*	0.19	0.43	0.17	0.20	30
	3.2	2.5	2.0					
1-year-old cultivated	*Elymus nutans*	*Polygonum viviparum*	*Nepeta coerulescens*	0.45	0.00	0.35	0.20	23
grassland A1	2.4	1.5	1.4					
3-year-old cultivated	*Elymus nutans*	*Poa pratensis*	*Koeleria cristata*	0.19	0.00	0.51	0.29	32
grassland A3	3.1	2.3	2.1					
4-year-old cultivated	*Elymus nutans*	*Koeleria cristata*	*Poa pratensis*	0.36	0.22	0.30	0.12	33
grassland A4	3.0	1.9	1.9					
6-year-old cultivated	*Elymus nutans*	*Koeleria cristata*	*Poa pratensis*	0.19	0.41	0.23	0.17	34
grassland A6	2.1	2.0	1.8					
8-year-old cultivated	*Poa pratensis*	*Koeleria cristata*	*Elymus nutans*	0.30	0.32	0.26	0.12	39
grassland A8	2.8	2.0	1.9					
10-year-old cultivated	*Elymus nutans*	*Koeleria cristata*	*Poa pratensis*	0.17	0.38	0.32	0.12	37
grassland A10	2.5	2.2	1.9					
12-year-old cultivated	*Elymus nutans*	*Poa pratensis*	*Poa crymophila*	0.20	0.23	0.46	0.12	35
grassland A12	2.2	2.1	1.8					
14-year-old cultivated	*Elymus nutans*	*Koeleria cristata*	*Poa pratensis*	0.20	0.13	0.41	0.25	29
grassland A14	3.1	1.7	1.5					
16-year-old cultivated	*Elymus nutans*	*Pedicularis kansuensis*	*Poa crymophila*	0.30	0.08	0.41	0.21	32
grassland A16	2.7	2.0	1.8					
18-year-old cultivated	*Elymus nutans*	*Poa pratensis*	*Koeleria cristata*	0.27	0.35	0.26	0.13	35
grassland A18	3.2	1.8	1.8					
Non-degraded	*Elymus nutans*	*Poa pratensis*	*Ligularia virgaurea*	0.29	0.31	0.33	0.07	41
grassland ND	2.3	1.5	1.3					

*G_λ, C_λ, L_λ, and F_λ, respectively, represent the proportion of comprehensive dominance of Grass, Cyperaceae, Legume, and Forb in the sampling plots.*

In the first 3 years after the cultivated grassland was built to replace the extremely degraded grassland, the proportion of comprehensive dominance of other functional groups except Cyperaceae increased. In the next 7 years, the proportion of dominance in Cyperaceae increased, while the proportion of other functional groups showed a fluctuating decrease trend. With the increase of recovery time, the dominance of Forb was close to 10%, while the dominance proportion of the other three functional groups was close to 30%, respectively (Table 1).

### Inter-Specific Association of Plant Communities Over Restoration Time

**FIGURE 2:**
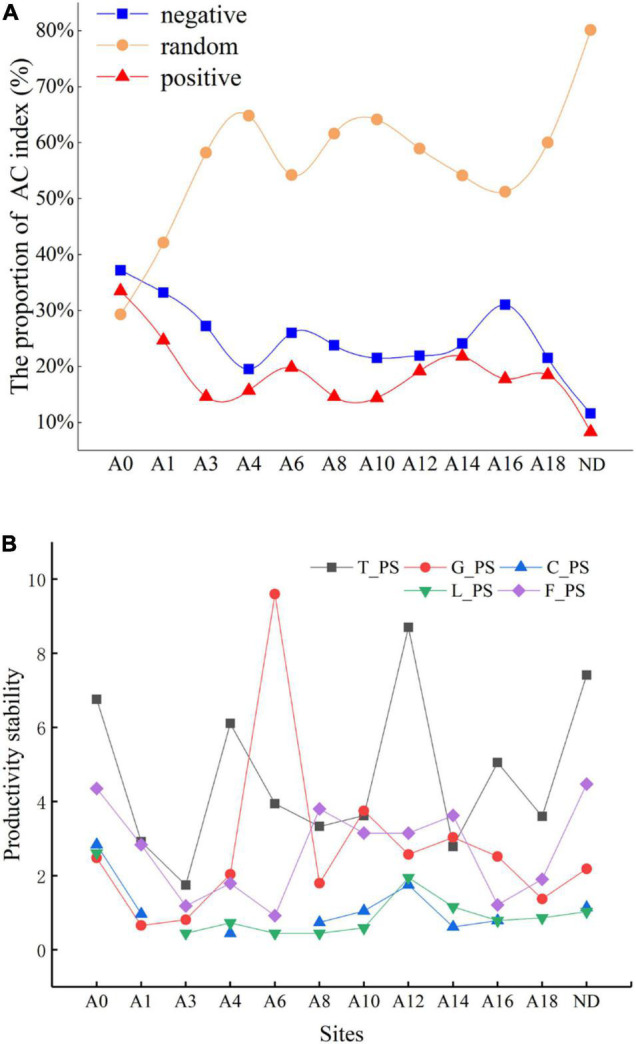
Changes in the proportion of inter-specific associations of the plant community **(A)** and stability index of artificial grassland productivity **(B)** in different years of construction. T_PS, G_PS, C_PS, L_PS, and F_PS, respectively, represent the stability of the total community, Grass stability, Cyperaceae stability, Leguminosae stability, and Forb stability. The number after the letter “A” indicates the restoration period of artificial grassland, A0 represents “black beach” and ND represents a non-degraded healthy alpine meadow.

We classified the inter-species relationships into three categories, positive inter-species association, negative inter-species association, and random inter-species association (Figure 2A). The proportion of species with random associations was higher than those of species with positive and negative associations in the plant community of all types of grasslands except “Black Beach.” The proportion of species with positive associations was slightly greater than those with negative associations in all types of grasslands. Throughout the restoration time, the proportions of species with both positive and negative associations generally tended to decrease and those of species with random associations tended to increase, although there were great fluctuations of inter-species relations after 4 and 10 restoration years.

The SNB of extremely degraded grasslands and cultivated grasslands planted for 1 year was greater than 4. The SNB values of other cultivated grasslands were all about 2–3, while the value of non-degraded grasslands was the minimum of 1.268. The complexity of plant interspecific association (species network density) was positive for non-degraded alpine meadow and cultivated grassland communities were positive in the 6th, 12th, and 16th years, while in the “Black Beach” degraded grassland it was negative. Plant interspecific association indicated that a few species highly influenced the entire network (Table 2).

**TABLE 2:** Network structure characteristics of cultivated grassland in different growing years.

Sites	SND	SNB
A0	−0.030	4.392
A1	−0.041	5.138
A3	−0.027	3.038
A4	−0.020	3.782
A6	0.011	3.03
A8	−0.021	2.706
A10	−0.019	2.501
A12	0.022	3.041
A14	−0.030	3.841
A16	0.000	2.708
A18	−0.003	3.214
ND	0.046	1.268

*SND, the complexity of plant interspecific association (network density); SNB, betweenness of plant interspecific linkage network (simplest network betweenness). The number after the letter “A” indicates the restoration period of artificial grassland, A0 represents “black beach,” and ND represents a non-degraded healthy alpine meadow.*

### Internal Driving Forces of Plant Community Stability Over Restoration Time

In the first 3 years of grassland restoration, the plant stability in the revegetated areas was significantly lower than that of the “Black Beach” degraded grassland (Figure 2B). The stability of Grass was increased after 6 years of restoration. The change in the productivity of the Forb was quite low. The similarity coefficients between the stability of Grass, Cyperaceae, Leguminosae, Forb, and the whole community were -0.158, 0.531, 0.659, and 0.368 respectively. The trend of stability of the whole community was similar to that of legumes, which was reflected by the greatest correlation among all plant groups.

The structural equation model was used to test the relationship and mediation between variables to study the influence mechanisms of plant community composition and diversity on grassland stability during the restoration process of alpine grassland. Since there was strong collinearity in the evenness and diversity of plant communities in different functional groups, eight factors including the evenness index of Grass (E-G), Cyperaceae (E-C), Leguminosae (E-L), Forb (E-F), Grass (H-G), Cyperaceae (H-C), Legumes (H-L), and Forb (H-F) were extracted and expressed as four principal components. We defined them as D-L, D-C, D-G, D-F, respectively, representing the diversity information of these four plant functional groups (Legume, Cyperaceae, Grass, Forb) (Table 3). Similarly, using the same analytical method, the total soil carbon (TC) and total soil nitrogen (TN), available phosphorus (AP), available potassium (AK), and soil moisture were extracted as two principal components. We defined them as soil nutrient and moisture, respectively (Table 4).

**TABLE 3:** The score matrix of the principal component analysis of the diversity of each functional group.

	Principal component			
	Legume	Cyperaceae	Grass	Forb
H_G				0.966
H_C		0.968		
H_L	0.972			
H_F			0.973	
E_G				0.979
E_C		0.984		
E_L	0.980			
E_F			0.985	
Factor	D-L	D-C	D-G	D-F

*H-G, H-C, H-L, H-F, respectively, represent Shannon–Wiener diversity index of Grass, Cyperaceae, Legume, and Forb in the sampling plots; E-G, E-C, E-L, E-F, respectively, represent Pielou’s evenness index of Grass, Cyperaceae, Legume, and Forb in the sampling plots; D-L, D-C, D-G, D-F, respectively, represent the diversity information of these four plant functional groups (Legume, Cyperaceae, Grass, Forb).*

**TABLE 4:** Principal component analysis score matrix of each soil property index.

	Principal component	
	Soil nutrients	Moisture
AP	0.830	
AK	0.894	
C	0.921	
N	0.872	
Soil moisture		0.983

*C, N, AP, and AK, respectively, represent the total soil carbon and total soil nitrogen, available phosphorus (AP), available potassium (AK), and soil moisture.*

**FIGURE 3:**
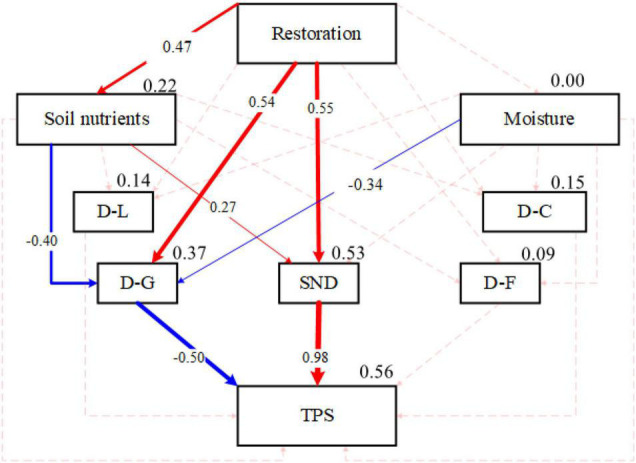
Structural equation model analysis results of community composition, network structure, and community stability path for the recovery years. D-L, D-C, D-G, and D-F, respectively, represent the diversity information of these four plant functional groups (Legume, Cyperaceae, Grass, Forb); TPS, stability of whole community; SND, the complexity of plant interspecific association (network density); Restoration represents human-induced revegetated grassland restoration. The boxes are the variables of the model. The solid line indicates a significant effect (*P* < 0.05); the dotted line indicates an insignificant effect (*P* > 0.05); Chi_square = 8.40 (*P* = 0.40); GF1 = 0.97; RMSEA = 0.03.

The results of the structural equation model indicated that compared with other functional groups, grass diversity had a more significant effect on the stability of the whole community during the restoration process of artificial grasslands (Figure 3). The restoration process of artificial grassland can directly affect or indirectly affect the species composition characteristics of the community (i.e., the network structure based on the interspecific relationship) by changing the soil nutrient conditions, and thus significantly affect the overall stability of the community.

## Discussion

Revegetation with cultivated grassland has been widely accepted as the best way to improve “Black Beach” degraded alpine grassland (Li et al., 2018), as it can improve the vegetation and soil of extremely degraded grassland in a short period (Li et al., 2014; Xu et al., 2019). We also found in this study that “Black Beach” can be intuitively changed within 3 years of plantation, and the proportion of excellent forages increased with restoration. From this perspective, we may conclude that the development of cultivated grassland can effectively curb the expansion of “Black Beach” on the QTP.

Interspecific association reflects the relationship between species pairs and their adaptability to the environment (Liu et al., 2019). In this study, we found that the proportion of species with positive and negative correlations was reduced in the cultivated grasslands over the restoration time. Identifying the positive or negative association of inter-species associations was meaningful to interpret the inter-specific relationships of different species in the plant community (Lou and Zhao, 2008). Although the mechanisms that regulate species interactions have not been fully elucidated, Bulleri et al. (2014) reported that the increase of time fluctuation in environmental stressors can enhance the absolute pressure level perceived by the interacting species. Some scholars found that inter-specific competition usually plays a destabilizing role (Loreau and De Mazancourt, 2013; Michalet and Pugnaire, 2016), while we can deduce that the relationship between species interactions will tend to be neutral as restoration proceeds in the cultivated grassland. Our findings are supported by Lou and Zhao’s (2008) study that mutual dependence and mutual competition in the plant community are greatly reduced and weakened in the long-term succession process. In later stages of restoration, each species occupies a narrower niche, so the plant community is stable with the harmonious coexistence of species. With the increase of restoration time, the inter-specific association of most species in cultivated grassland tends to be zero, which was highly related to the natural performance of plant communities. After 4 years of restoration, the number of unconnected species in the community decreased, indicating that the community was susceptible to external interferences (such as human disturbance and livestock feeding) and tended to reverse succession.

Plant composition is the most intuitive feature that determines the appearance, structure, and function of the grassland community, and even its successional direction (Wei et al., 2011; Guo et al., 2019). We hypothesize that the composition and structure of species may be the internal driving force for the change of community stability. Most experiments showed that exaggerated species diversity can promote increased stability of productivity over time (Hautier et al., 2014), for example, Río et al. (2017) reported from a study on European forests that changes in community composition can change the temporal stability of community productivity, and external and internal factors affecting the species composition may impact the stability of community productivity (Gross et al., 2014). With the progress of related research, scientists have also emphasized the important role of species composition and inter-specific relationships (Isbell et al., 2009; Klanderud, 2010; Loreau and De Mazancourt, 2013; de Mazancourt et al., 2013). In theoretical ecology, traditional research based on dynamic stability and numerical simulation has not found a unified answer to explain the impact of network architecture on community persistence (Rohr et al., 2014). All of these can be used as evidence to support the importance of species composition and interspecific relationships in influencing community stability and even ecosystem services and functions (Johnson et al., 2008; Maselli et al., 2013; Pellissier et al., 2018; Zeeman et al., 2019). Social network analysis methods based on interspecies relationships may provide a quantitative means for exploring the characteristics of community structure for future studies of grassland communities on the QTP.

## Conclusion

With the extension of planting time, the relationship between species interactions of cultivated grasslands for replacing “Black Beach” on the QTP will become more neutral. In the present study, we found that the complexity of plant interspecific association (species network density) had more influence on community stability with the extension of recovery time. The novel method of quantifying the structural characteristics of a community for gauging its stability can provide a quantitative way to explore the characteristics of community structure. The plant species network that is based on species interaction is needed to support the applicable and efficient revegetation actions, for which restoring the severely degraded alpine grasslands on the QTP and even worldwide is essential.

## Data Availability Statement

The original contributions presented in the study are included in the article/supplementary material, further inquiries can be directed to the corresponding authors.

## Author Contributions

SW and SL: conceptualization. SW: methodology, software, validation, formal analysis, resources, data curation, writing—original draft preparation, and visualization. SW, XG, and YX: investigation. SD and LW: writing—review and editing and funding acquisition. SD and KW: supervision. SD and QD: project administration. All authors contributed to the article and approved the submitted version.

## Conflict of Interest

The authors declare that the research was conducted in the absence of any commercial or financial relationships that could be construed as a potential conflict of interest.

## Publisher’s Note

All claims expressed in this article are solely those of the authors and do not necessarily represent those of their affiliated organizations, or those of the publisher, the editors and the reviewers. Any product that may be evaluated in this article, or claim that may be made by its manufacturer, is not guaranteed or endorsed by the publisher.
